# Supplementary material for: Thoracic UltrasONOgraphy Reporting: The TUONO Consensus
Source: Diagnostics (Basel). 2023 Apr 25;13(9):1535. doi: 10.3390/diagnostics13091535 (PMC10177560; doi:10.3390/diagnostics13091535)
Supplement: Supplementary file 1 [file diagnostics-13-01535-s001.zip › diagnostics-2341576-supplementary.pdf]

Supplemental materials

Panel S1: First round – MCQ

Section 1
Modified-Delphi first round is aimed to investigate the possibility of reaching a consensus over the essential characteristics of thoracic ultrasound reporting.

Question 1: center code

Section 2
Topic 1: localization of signs
Context
In literature, several models of examination of the thorax have been reported in order to define localization and/or quantify LUS findings.

Question 1: Which is the best model to localise LUS sings?
Question 2: In a free text report (FTR), regarding the localization of the findings:
1. it is preferable to refer to previously defined thoracic areas
2. it is preferable to use references such as anterior, posterior, superior, inferior, apical, basal, lateral, right, left, bilateral, etc.
3. Other

Question 3 In addition to indicating an area, a more precise location of findings with anatomical landmarks such as *on the right hemiclavear at the 3rd intercostal space* or *on the middle axillary at the fifth intercostal space*:
1. is required for all the findings
2. is necessary for some findings such as the lung point in case of PNX or small subpleural thickenings
3. is never necessary
4. Other

Section 3
Topic 2: Normal areas, pathological areas and unexamined areas
Context
Differently from chest XR or CT which examines the entire chest, LUS can only be performed in selected areas. Therefore, the report of a pathological finding (e.g. right basal thickening) does not assure that all the areas have been explored.

Question 4 - Is it important to indicate which areas were NOT examined in LUS report?
1. Yes
2. No
3. Other

Question 5 - If it is reported that all the areas have been examined, those not described as pathological:
1. should be automatically considered normal without further explanation
2. should be described as normal
3. other

Question 6 - Is it important to indicate whether the pleurae have been examined?
1. Yes
1. No
2. Other

Question 7 - The characteristics of the pleura:
1. should be described even when normal (e.g. sliding present, normal aspect)
2. should be described just in case of abnormalities (absent sliding, presence of lung points, irregular appearance, etc.), otherwise they should be considered as normal
3. other

Topic 3: ultrasound signs / findings
Context
Several LUS signs have been reported in literature. The analysis of collected reports for Tuono 2 study showed some of them are seldom used.

Question 8 – Which signs do you consider as keystones in daily clinical practice?
1. A lines
2. B lines
3. Bat sign
4. Seashore sign
5. Shred sign
6. Consolidation
7. Curtain sign
8. Pleural effusion
9. Sinusoid sign
10. Quad sign
11. Stratosphere sign
12. Lung point
13. Lung sliding
14. Lung pulse
15. Static air bronchogram
16. Dynamic air bronchogram

Question 8a - Do you think there are other important LUS signs and/or findings?

Question 9 - In Lichtenstein's *BLUE Protocol* (Chest 2008) LUS profiles were described on the basis of the presence and localisation of pathognomonic signs combined with the presence or absence of pleural sliding. Which profile should be used to examine and report LUS?
1. A Profile (A lines with sliding present)
2. A' Profile (A lines with absent sliding)
3. B Profile (B lines with sliding present)
4. B' Profile (B lines with absent sliding)
5. C Profile (consolidations in anterior zones)
6. PLAPS Profile (Postero Lateral Alveolar or Pleural Syndrome)
7. None of the above

Question 10 - To describe an area or region:
1. it is preferable to use ultrasound signs (see question 8)
2. it is preferable to use ultrasound profiles (see question 9)
3. both signs and ultrasound profiles can be used
4. other

Topic 4: quantification of pathological findings
Question 11 - In addition to the presence of a given finding, should it be described in terms of quantification or severity in LUS report?
1. Yes
2.No
3.Other

Question 12 - The B lines in the examined area:
1. should be reported in a semi-quantitative manner (e.g. *some, few, moderate, many*)
2. should be quantified by counting their number
3. should be quantified as *separate* or *bundled*
4. should not be quantified; it is satisfactory to report their presence
5. other

Question 13 – Regarding pleural effusion:
1. it should not be quantified; it is satisfactory to report their presence
2. it should be quantified in a semi-quantitative manner (e.g. *minimal, mild, moderate, severe, massive*)

3. it should be quantified (e.g. measuring its extension in cm in the point of major thickness, counting the intercostal spaces where it is detectable)
4. it should be quantified by estimating the volume in milliliters
5. other

Question 14 - Regarding the description of pleural effusion:

1. it should always be described in a qualitative manner (e.g *anechoic*, *hypoechoic*, *corpuscular*)
2. the quality of an effusion (hypoechoic, corpuscular, buried, sacked) should be described only if the effusion is not anechoic/hypoechoic and/or free
3. other

Question 15 – Regarding consolidation:

1. it should not be quantified; it is satisfactory to report its presence
2. it should be quantified in a semi-quantitative manner (e.g. *small*, *extended*)
3. it should be quantified (e.g. measuring its extension in cm in the point of major thickness, counting the intercostal spaces where it is detectable)
4. it should be quantified by estimating its volume in milliliters
5. other

Section 6

Topic 5: The Scores

Context

The Lung Ultra Sound Score (LUSS) identifies 4 progressive stages of lung aeration loss (0 = normal; 1 = separate B lines; 2 = coalescent B lines; 3 = consolidation). The LUSS has been proposed as a semi-quantitative method of lung aeration by assigning a score from 0 to 3 in 12 lung areas and then assuming a value from 0 (all normal areas) to 36 (all consolidated areas).

Question 16 - Is it useful to calculate and state LUSS in the report?

1. Yes
2. No
3. Other

Section 7

Topic 6: Terminology to describe the various zones

Question 17 - Describing lung zones:

1. it is better to list the ultrasound findings (e.g. B lines) or the ultrasound profiles (profile A '), possibly writing the interpretation / diagnosis in the Conclusions Section

2. it is better to write directly the diagnosis reached with the interpretation of the report (eg: "interstitial edema in the x-zone")

3.other

Question 18 - How would you describe an area with numerous B lines? More than one answer possible.

1. area with *numerous B lines*
2. area with signs of *pulmonary congestion*
3. area with *water overload*
4. area with *interstitial edema*
5. area with *interstitial disease*
6. all of the above

Section 8

Topic 7: conclusions section

Question 19 - The conclusions section:

1. should report US appearance (e.g. *B lines, diffuse interstitial disease*) but not necessarily the clinical interpretation
2. should report both US appearance and clinical hypothesis (e.g. *interstitial disease compatible with pulmonary edema or ARDS or pulmonary fibrosis*)
3. should report clinical diagnosis but not necessarily LUS findings
4. is not necessary, since the findings have been previously described

5. other

Question 20 - The conclusions section should be:

1. a free text box to allow the description of elements not included in the structured report
2. structured, according to different options
3. other

Section 9

Topic 8: Additional Information

Question 21 - In the report what additional information should be present?

21 a) Diagnosis of the patient (e.g. *respiratory failure, trauma*)

1. Yes
2. No
3. Other

21 b) Purpose of the investigation (e.g. monitoring, follow-up, clinical suspicion)

1. Yes
2. No
3. Other

21 c) Patient position

1. Yes
2. No
3. Other

21 d) Technical difficulties (if patient position reported)

1. Yes
2. No
3. Other

21 e) Type of probe used

1. Yes
2. No
3. Other

21 f) type of ventilation

1. Yes
2. No
3. Other

21 g) Changes in findings after modification of ventilatory setting (e.g. recruitment maneuver)

1. Yes
2. No
3. Other

21 h) Comparison with previous exams

1. Yes
2. No
3. Other

21 i) Diagnostic path (e.g. *suspected pneumothorax to be confirmed by CT scan*)

1. Yes
2. No
3. Other

21 l) Therapeutic path (e.g., *indication to pleural drainage*)

1. Yes
2. No

3. Other

Section 10
Topic 9: Other relevant topics related to thoracic ultrasound reporting

Question 22 - What topics not present in this questionnaire do you think it is important to report?

Panel S2 TUONO REPORTING TOOL: a proposal for LUS structured form

Name______________________________Surname______________________________Date of birth _____

Clinical scenario/framework: ______________________________

Purpose of examination: □ screening □ monitoring □ procedural

Position: □ supine □ prone □ sitting □ right lateral □ left lateral

Breathing/ ventilation: □ Spontaneous breathing □CPAP □NIV □ Invasive ventilation

NOTES

ZONES and FINDINGS

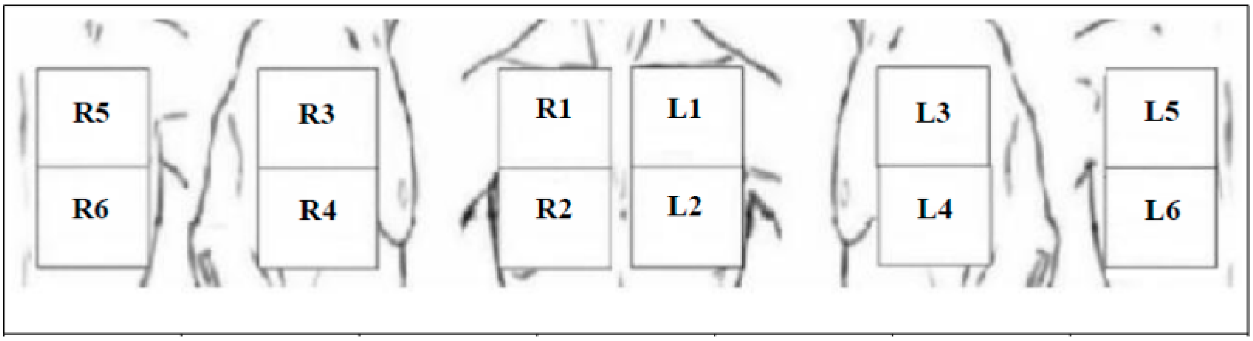

Normal	B lines: well-spaced	B2 lines: coalescent	Tissue-like consolidation	Coalescent B lines & Effusion	Tissue-like consolidation & Effusion	Effusion
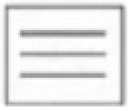	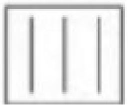	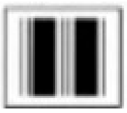	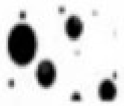	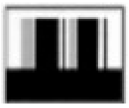	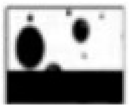	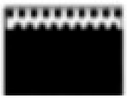
LUSS point = 0	LUSS point = 1	LUSS point = 2	LUSS point = 3	LUSS point = 2	LUSS point = 3	/
**LUNG ULTRASOUND SCORE (LUSS) =**						
RIGHT PLEURA				LEFT PLEURA		
Posterior<br>□ Normal<br>□ Pathological	Lateral<br>□ Normal<br>□ Pathological	Anterior<br>□ Normal<br>□ Pathological		Anterior<br>□ Normal<br>□ Pathological	Lateral<br>□ Normal<br>□ Pathological	Posterior<br>□ Normal<br>□ Pathological

CONCLUSIONS

Example: the graphic display of LUS features as pictograms (A) and the corresponding sonographic images

A)

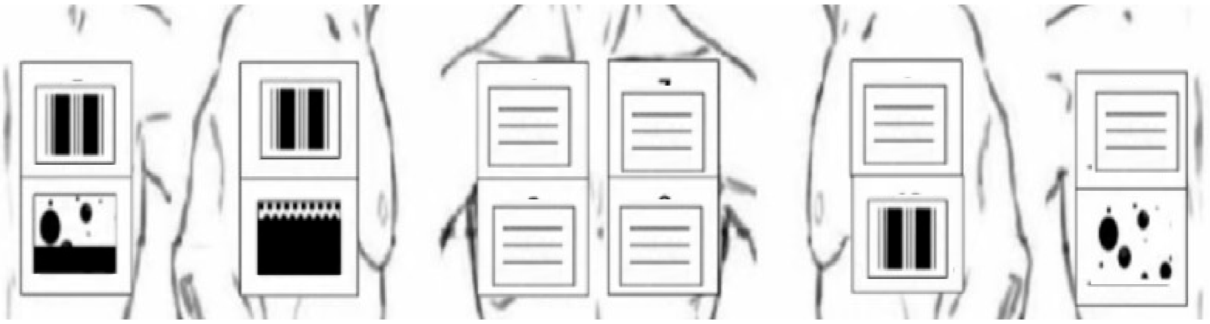

B)

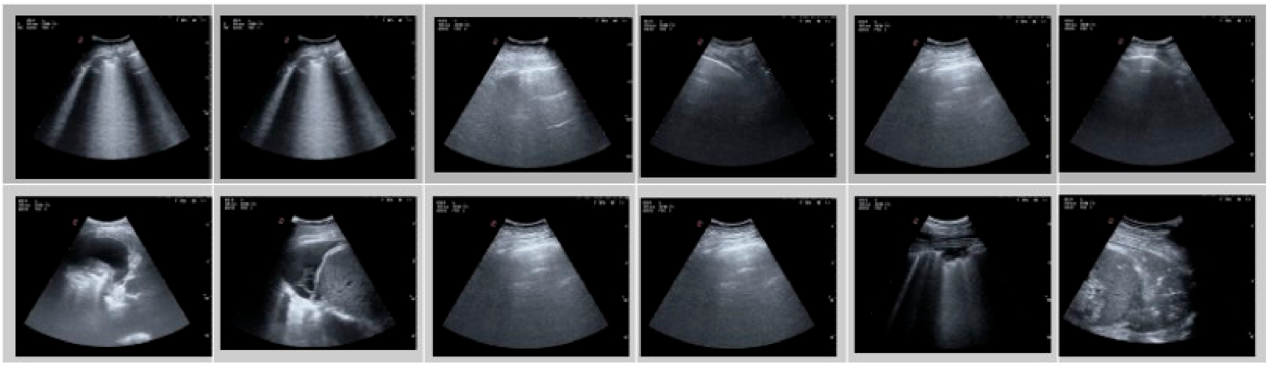

t

Panel S3: List of experts and clinicians participating to the Consensus Conference

-Andrea Bottazzi, Department of Anesthesia and Critical Care Medicine, IRCCS Policlinico San Matteo Foundation, Pavia, Italy
-Vittoria Bartolini, Intensive Care Unit 1, Santa Maria Annunziata Hospital, Bagno a Ripoli, Firenze, Italy;
-Martina Martinelli, Intensive Care Unit 1, Santa Maria Annunziata Hospital, Bagno a Ripoli, Firenze, Italy;
-Romano Giuntini Intensive Care Unit S. Giuseppe Hospital Empoli, Florence, Italy
-Valentina Ormas, Department of Anesthesiology and Intensive Care, ASST Lecco, Lecco, Italy; ---
-Andrzej Michal Bet, Department of Anesthesiology and Intensive Care, ASST Lecco, Lecco, Italy;
-Riccardo Giudici, Department of Anesthesia and Intensive Care Medicine, ASST GOM Niguarda, Milan, Italy;
-Loredana Faraldi, Department of Anesthesia and Intensive Care Medicine, ASST GOM Niguarda, Milan, Italy;
-Andrea Ciani, Intensive Care Unit, S.S. Cosma e Damiano Hospital, Pescia, Pistoia, Italy;
-Chiara Ladu, Intensive Care Unit, S.S. Cosma e Damiano Hospital, Pescia, Pistoia, Italy;
-Nunzio Dario Cascio, Intensive Care Unit, Mauriziano "Umberto I" Hospital, Torino, Italy;
-Mauro Gallo, Intensive Care Unit, Mauriziano "Umberto I" Hospital, Torino, Italy;
-Maurizio Saini, Department of Anesthesiology and Intensive Care, Desio Hospital, ASST Brianza, Desio, Italy; Marta Zanola, Postgraduate School of Anaesthesiology and Intensive Care, Università degli Studi di Milano Bicocca, Milano, Italy;
-Paola Previtali, Intensive Care Unit, Fondazione IRCCS Istituto Nazionale dei Tumori, Milano, Italy;
-Luca Fumagalli, Intensive Care Unit, Fondazione IRCCS Istituto Nazionale dei Tumori, Milano, Italy;
-Luigi Barile, Department of Anesthesiology and Intensive Care, ASO Santa Croce e Carle, Cuneo, Italy; Marco Pezzuto, Department of Anesthesiology and Intensive Care, General Regional Hospital "F. Miulli", Acquaviva delle Fonti, Bari, Italy;
-Luigi Pisani, Department of Anesthesiology and Intensive Care, General Regional Hospital "F. Miulli", Acquaviva delle Fonti, Bari, Italy;
-Alberto Guadagnucci, Department of Anesthesiology and Intensive Care, Ospedale del Cuore, Fondazione Monasterio, Massa, Italy
-Cristina Alessandrini, Department of Anesthesiology and Intensive Care, Ospedale del Cuore, Fondazione Monasterio, Massa, Italy;
-Sara Casarotti Todeschini, Department of Anesthesiology and Intensive Care, Santa Maria del Prato Hospital, AULSS 1 Dolomiti, Feltre, Belluno, Italy;
-Monica Verri, Department of Anesthesiology and Intensive Care, Santa Maria del Prato Hospital, AULSS 1 Dolomiti, Feltre, Belluno, Italy;
-Gianna Cestelli, Department of Anesthesiology and Intensive Care, Santa Maria Nuova Hospital, AUSL Toscana Centro, Firenze, Italy;
-Maria Teresa Tanzi, Department of Anesthesiology and Intensive Care, Santa Maria Nuova Hospital, AUSL Toscana Centro, Firenze, Italy;
-Giovanna Branca, Department of Anesthesiology and Intensive Care, Ospedale del Delta, AUSL Ferrara, Lagosanto, Ferrara;
-Erminio Righini, Department of Anesthesiology and Intensive Care, Ospedale del Delta, AUSL Ferrara, Lagosanto, Ferrara;
-Vieri Parrini, Department of Anesthesiology and Intensive Care, Mugello Hospital, AUSL Toscana Centro, Borgo San Lorenzo, Firenze, Italy;
-Giuliano Michelagnoli, Department of Anesthesiology and Intensive Care, Mugello Hospital, AUSL Toscana Centro, Borgo San Lorenzo, Firenze, Italy;

-Valentina Chiarini, Intensive Care Unit and Territorial Emergency, Maggiore Hospital, AUSL di Bologna, Bologna, Italy;
-Enrico Ferri, Intensive Care Unit and Territorial Emergency, Maggiore Hospital, AUSL di Bologna, Bologna, Italy;
-Tamara Biscioni, Department of Anesthesiology and Intensive Care, Felice Lotti Hospital, AUSL Toscana Nord Ovest, Pontedera, Pisa, Italy;
-Dino Di Pasquale, Department of Anesthesiology and Intensive Care, Felice Lotti Hospital, AUSL Toscana Nord Ovest, Pontedera, Pisa, Italy;
-Fabio Piccirillo, Department of Anesthesiology and Intensive Care, IRCCS Humanitas Research Hospital, Milan, Italy;
-Elena Costantini, Department of Anesthesiology and Intensive Care, IRCCS Humanitas Research Hospital, Milan, Italy;
-Andrea Della Selva, Department of Anesthesiology and Intensive Care, Michele e Pietro Ferrero Hospital, Verduno, Cuneo, Italy;
-Luigi Junior Laricchia, Department of Anesthesiology and Intensive Care, Michele e Pietro Ferrero Hospital, Verduno, Cuneo, Italy;
-Michele Casalis, Intensive Care Medicine, Piombino Hospital, AUSL Toscana Nord Ovest, Piombino, Livorno, Italy;
-Alessio Demi, Intensive Care Medicine, Piombino Hospital, AUSL Toscana Nord Ovest, Piombino, Livorno, Italy
